# Stability and Chemical Conversion of the Purified Reference Material of Gymnodimine-A under Different Temperature and pH Conditions

**DOI:** 10.3390/toxins14110744

**Published:** 2022-10-29

**Authors:** Guixiang Wang, Jiangbing Qiu, Aifeng Li, Ying Ji, Zhixuan Tang, Philipp Hess

**Affiliations:** 1 College of Environmental Science and Engineering, Ocean University of China, Qingdao 266100, China; 2 Key Laboratory of Marine Environment and Ecology, Ocean University of China, Ministry of Education, Qingdao 266100, China; 3 Ifremer, PHYTOX Research Unit, F-44000 Nantes, France

**Keywords:** gymnodimines (GYMs), stability, chemical conversion, liquid chromatography–high resolution mass spectrometry (LC-HRMS), certified reference materials (CRMs)

## Abstract

Gymnodimines (GYMs) are a group of fast-acting phycotoxins and their toxicological effects on human beings are still unclear due to the lack of sufficiently well-characterized large quantities of purified toxins for toxicology studies. In this study, a certified reference material (CRM) of GYM-A was prepared from the dinoflagellate *Karenia selliformis*, followed by multi-step chromatography separation and purification. Subsequently, the stability of GYM-A in methanolic media was evaluated at different temperature (−20, 4, and 20 °C) and pH (3, 5, and 7) conditions for 8 months, and the conversion products of GYM-A were explored by liquid chromatography–high resolution mass spectrometry (LC-HRMS). The results show that the stability of GYM-A decreased with increasing temperature and pH values. The GYM-A was stable during storage at −20 °C regardless of pH, but it decreased rapidly (81.8% ± 9.3%) at 20 °C in pH 7 solution after 8 months. Moreover, the concentrations of GYM-A did not significantly change at all temperatures in solutions with pH 3 (*p* > 0.05). It is recommended that GYM-A should be stored at low temperature (≤−20 °C) and pH (≤3) conditions for long-term storage in aqueous methanolic media. In addition, two conversion products of GYM-A, tentatively named as GYM-K (*m*/*z* 540) and GYM-L (*m*/*z* 524), were identified in the samples stored at high levels of pH and temperature. Based on the LC-HRMS data, the hypothetical chemical structures of both converting derivatives were proposed. A useful strategy for long-term storage of GYM-A CRM in aqueous methanolic media was suggested and two hypothesized conversion products of GYM-A were discovered in this study.

## 1. Introduction

Gymnodimines (GYMs) are a group of lipophilic marine toxins, which are referred to as the Cyclic Imine (CI) group of toxins due to their cyclic imine structure. Since the first discovery of GYMs in oysters from New Zealand in 1994 [1], GYMs have been detected frequently in many countries worldwide [2,3,4,5,6]. Based on the acute toxicity in mice by intraperitoneal injection, GYMs are also known as “fast-acting toxins” [7]. Although the toxic mechanism of GYMs on the acetylcholine receptor (AChR) antagonism and the neuromuscular system were reported in some previous studies [8,9,10], knowledge of their toxicological effects and chronic toxicity to human health is still very limited. 

**Figure 1:**
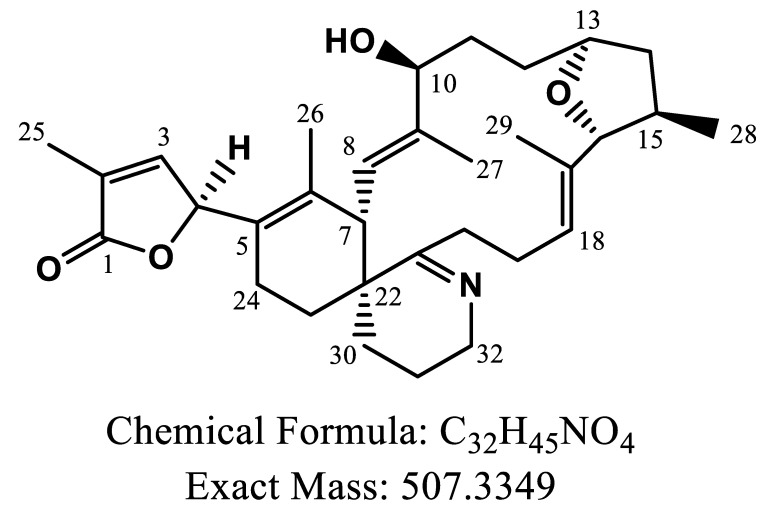
Chemical structure and exact mass of gymnodimine-A.

The chemical structure of GYM-A was first elucidated by nuclear magnetic resonance (NMR) in 1995 [1]; then, another seven analogues of GYM-A were discovered and identified successively (Figure 1), including GYM-B/C [11,12], 12-methyl GYM-A/B [13,14], GYM-D [15], 16-desmethyl GYM-D and GYM-E [16]. The GYM-A is mainly produced by the dinoflagellate *Karenia selliformis* isolated from several regions such as New Zealand, Tunisia and China [17,18,19]. Generally, the free form of GYM-A could be accumulated and metabolized to form fatty acid esters in shellfish exposed to *K. selliformis* or dissolved toxins in seawater under laboratory conditions [20,21], and the majority of GYMs were stored as the acylated form (>90%) in field shellfish samples [22], suggesting that these metabolites cannot be neglected during assessment due to the risk of GYMs to human beings. To discover new metabolites of GYMs, Varriale et al. developed a novel data-dependent acquisition-based approach of liquid chromatography–high resolution mass spectrometry (LC-HRMS), and successfully found five new hypothetical analogues of GYMs, namely GYM-F, -G, -H, -I and -J [23].

Stability evaluation is an essential process to guarantee the quality and concentration of certified reference materials (CRMs). The stabilities of paralytic shellfish toxins (PSTs) [24], diarrhetic shellfish toxins (DSTs) [25] and azaspiracids (AZAs) [26] have been reported under various pH and temperature levels. Presently, the National Research Council Canada (NRC) can supply the CRM of GYM-A; however, the stability of GYM-A during long-term storage is still incomprehensive, and the technical report showed that GYM-A was degraded significantly at 37 °C in a long-term study after 6 months [27], but the influence of medium pH on its stability was not reported, which is an important factor for GYM-A long-term stability. Storage conditions, such as temperature, the pH of solvent medium and duration, are responsible for toxin stability, e.g., PSTs and their hydroxyl metabolites (M-toxins) were more stable at a lower pH (3) and temperature (<−20 °C) [24,25,28], while azaspiracid-1 degraded rapidly under acidic conditions at 37 °C within 10 min [26]. Recently, a non-target analysis using LC-HRMS was developed for the stability assessment of multiple toxins, and no instability was measured for marine algal toxins stored below 4 °C [29]. The GYM-A was relatively stable in the acidulated culture media (seawater) at pH 5 within 7 days, while it obviously degraded under alkaline and neutral conditions in our previous study [30]. To our knowledge, a specific profile on the stability and conversion of GYM-A under different storage conditions is still unavailable now. Therefore, it is urgent to evaluate the stability of GYM-A to provide some satisfactory strategies for its long-term storage.

In this study, the reference material of GYM-A was prepared from the cultures of the GYM-producing dinoflagellate *K. selliformis*. Then, the long-term stability of GYM-A was evaluated under various temperature (−20, 4, and 20 °C) and pH levels (3, 5, and 7) during 8-month storage. Based on analysis of two unknown compounds related to GYM-A using LC-HRMS, the chemical structures of two conversion products of GYM-A were hypothesized in this work. 

## 2. Results and Discussion

### 2.1. Homogeneity Assessment

Homogeneity assessment is the premise of stability studies, it is important that the material within each unit is uniform to ensure accurate quantification of samples. In this study, large-scale cultures of GYM-producing dinoflagellate were conducted in photobioreactors, and sufficient content of GYM-A was prepared by multi-step chromatographic separation. Then, ten bottles selected randomly from the whole batch purification were measured by LC-MS/MS in triplicate. Concentrations of GYM-A in these samples are shown in Table S1. The normalized relative observed analytical values ranged from 0.9 to 1.1 (Figure S1); specifically, the maximum deviation for both the lowest and the highest quantitative result were 9.3% and 10.0%, indicating that the GYM-A basically had a good homogeneity. The ANOVA results show that the GYM-A concentrations did not significantly vary between and within bottles (*p* > 0.05) (Table S2). Thus, the purified GYM-A bottled solutions were considered sufficiently homogeneous and met the requirements of stability studies. Moreover, the GYM-A purity was assessed by a mass spectrometry full-scan test (Figure S2) and qNMR analysis (Figure S3, Table S3), and the samples were detected with high purity (>95%).

### 2.2. Stability Assessment

The assessment of the solvent volume over the time period of the experiment and between different temperatures is very important for the evaluation of the material’s stability. Thus, the solvent weight was assessed using an analytical balance before each test, and then converted into solvent volume. The results show that the solvent volume did not significantly change in the first two-month period (*p* > 0.05), but significantly changed (*p* < 0.05) after four months (Table S4–6). The concentrations of GYM-A after four months were weighted to consider any change in the solvent volume.

**Figure 2:**
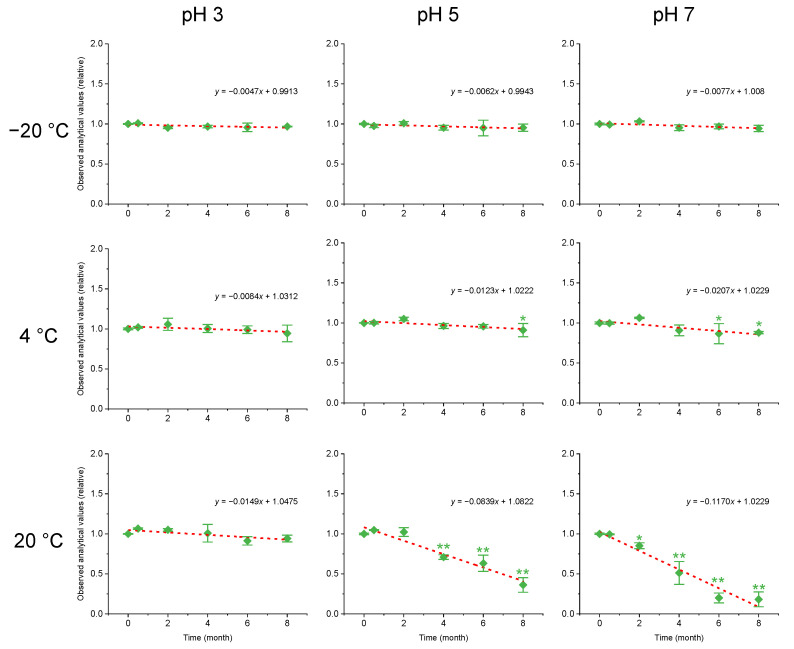
Variations of the relative concentrations of GYM-A under different temperature and pH conditions for 8 months. The dashed line represents the linear regression. * and ** indicate statistically significant difference compared with the initial status at *p* < 0.05 and *p* < 0.01, respectively.

The variations of GYM-A under different storage conditions for eight months are shown in Figure 2. The results demonstrate that the GYM-A stability was significantly affected by temperature and pH. Specifically, the GYM-A concentration did not significantly change stored at −20 °C or pH 3 during 8-month storage (*p* > 0.05), while it diminished significantly at pH 5 and 7 after 8 months at 4 °C (*p* < 0.05). Under the condition of 20 ℃, an extremely significant decrease in GYM-A concentration occurred at pH 5 and 7 after 8 months (*p* < 0.01), of which 14.8 ± 3.7% decreased (*p* < 0.05) within the first two-month period and 81.8 ± 9.3% decreased (*p* < 0.01) after 8 months at pH 7, while it showed no obvious decline under the treatment of pH 3 (*p* > 0.05). The *t*-test results (Table 1) show that GYM-A was stable when it was stored at −20 °C or pH 3 after 8 months (*p* > 0.05), while it was unstable at pH 5 and 7 under the temperature of 4 or 20 °C after 8 months (*p* < 0.05), indicating that the stability of the GYM-A was considerably affected by high-temperature and pH conditions.

**Figure 3:**
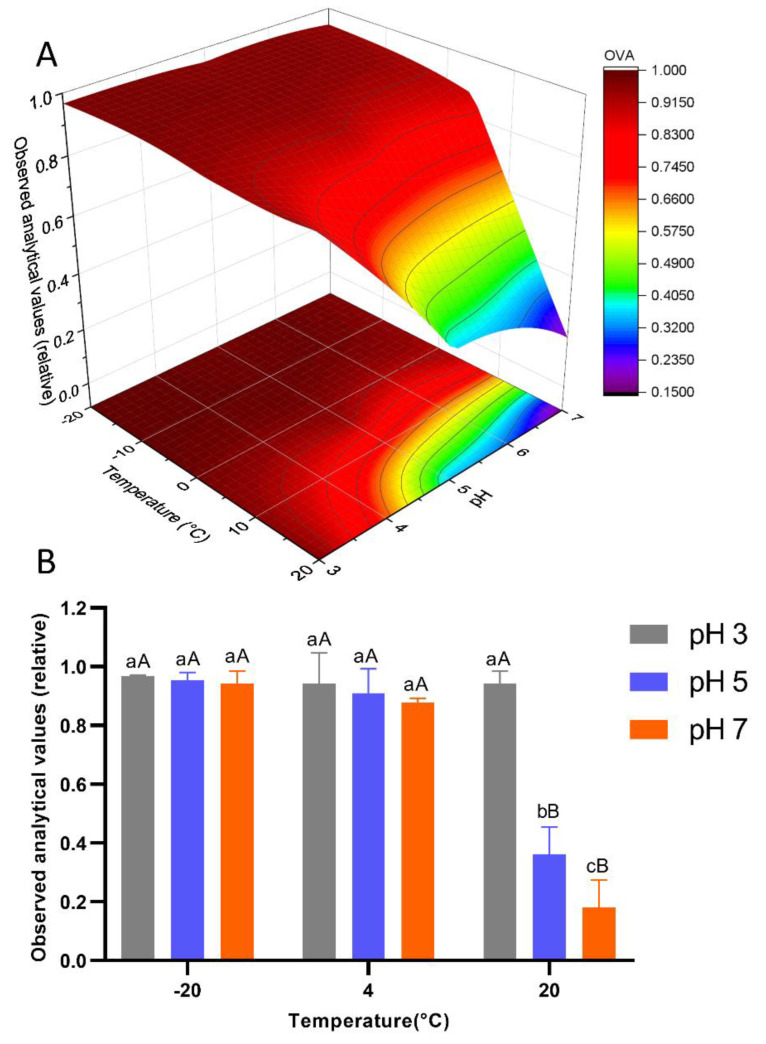
Observed relative concentrations of GYM-A under different temperature and pH conditions after 8-month storage: (**A**) three-dimensional contour map; OVA represents relative observed analytical values. (**B**) two-way ANOVA of different treatments. Different lowercase letters show significant difference among different pH conditions (*p* < 0.05), and different capital letters show significant difference among different temperatures (*p* < 0.05).

To better visualize the changes in GYM-A stability under different temperature and pH conditions, the data obtained at the timepoint after 8 months were plotted as a three-dimensional (3D) contour map, shown in Figure 3A. Overall, it displayed a waterfall shape that was relatively flat in the range of −20 to 0 °C, while it fell off a cliff in the range of 0 to 20 °C at a high pH value. The concentrations of GYM-A under different treatments after 8 months were analyzed by two-way ANOVA, and the results are shown in Figure 3B. At 20 °C, the higher the pH value, the lower the GYM-A concentration (*p* < 0.05), indicating that GYM-A was more stable in the acid medium. However, this phenomenon faded away when the temperature dropped below 4 °C, and the GYM-A concentration was no longer affected by pH (*p* > 0.05). In addition, two-way ANOVA results show that temperature and pH synchronously affected the stability of GYM-A (*p* < 0.05) during the long-term storage.

Different marine phycotoxins usually show different sensitivity to pH and temperature during storage, e.g., azaspiracid-1 (AZA1) degraded rapidly under acidic conditions at 37 °C within 10 min, while domoic acid (DA) was stable at pH 3–9 after heating at 121 °C for 30 min [26,31]. In the present study, GYM-A was stable at −20 °C regardless of pH over the whole monitoring period, but significantly decreased at pH 5 and 7 at 20 °C. Paralytic shellfish toxins (PSTs) exhibited similar stable characteristics to GYM-A, which were more stable at a lower pH (pH 3–4) and temperature (−35 °C), but decreased universally at a higher pH during storage at 25 °C [24]. Meanwhile, their hydroxyl metabolites (M-toxins) were also very sensitive to high pH and temperatures [28]. Moreover, PSTs significantly decreased in scallop tissues at −20 °C after 6-month storage [32], suggesting that the toxin stability was also affected by the storage medium. Notably, however, almost all toxins were more stable at lower temperatures, probably because low temperatures limited the activities of relevant enzymes or catalysts, which are essential to the chemical conversion of toxins. Furthermore, Jackson et al. pointed out that pinnatoxin-A (PnTx-A) showed remarkable chemical stability in neutral and strongly acidic aqueous solution due to kinetic, rather than thermodynamic, factors [33]. Hence, GYM-A was hypothesized to be thermodynamically stable at low temperatures (<−20 °C) regardless of pH. Effects of pH on toxin stability may not only include effects on the activities of enzymes or catalysts but may also be related to the chemical properties of the toxins [26], suggesting that the degradation or conversion of toxins should be performed under appropriate pH conditions. From the results in this study, acidic conditions did not result in the conversion of GYM-A, which was consistent with the fact that GYM-A was more stable in acidic seawater in our previous study [30]. Therefore, it is recommended that GYM-A should be dissolved in acidic medium (pH ≤ 3) and stored in the condition of ≤−20 °C during long-term storage.

### 2.3. Hypothetical Structure of the Converting Products of GYM-A 

**Figure 4:**
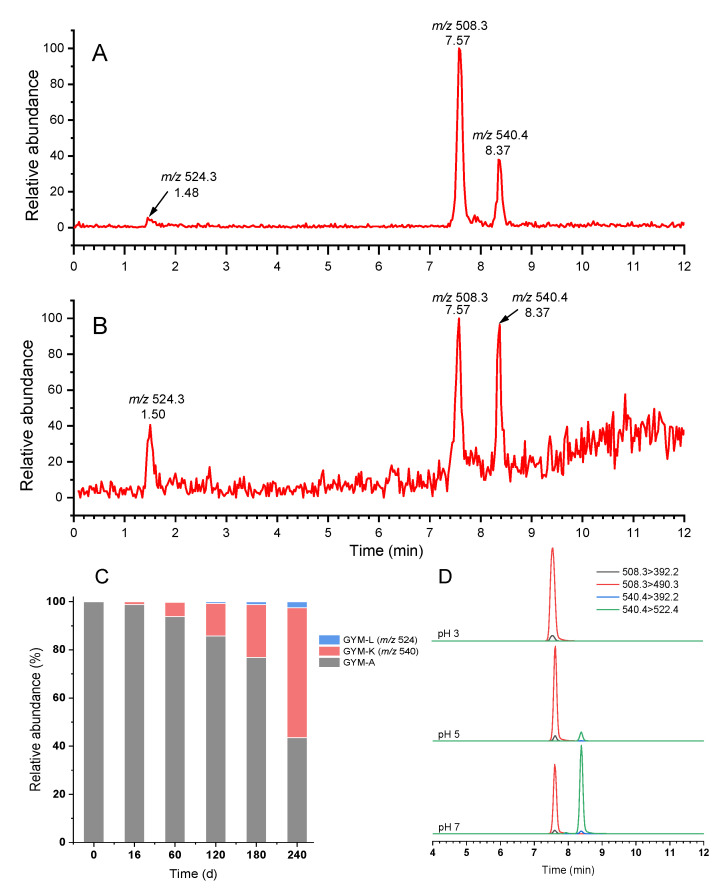
Non-target screening and validation of conversion products: precursor ion scan chromatograms of the product ion at *m*/*z* 392.2 (**A**) and *m*/*z* 136.1 (**B**); the relative abundance of GYM-A, GYM-K (*m*/*z* 540) and GYM-L (*m*/*z* 524) at different time points (20 ℃, pH 7) (**C**); LC-MS/MS chromatograms for GYM-A and GYM-K at different pH under 20 ℃ after 8 months (**D**).

The stability experiment showed that the concentration of GYM-A decreased sharply by approximately 82% stored at 20 °C in pH 7 after 8 months. Accordingly, it was suspected that GYM-A might have been converted to some unknown products. Therefore, the unknown compounds were explored by LC-MS/MS using the precursor ion scan mode with the product ions of *m*/*z* 392.2 and 136.1. Two obvious peaks with the retention times (RTs) of 7.57 min and 8.37 min present in the chromatogram of *m*/*z* 392.2 (Figure 4A), in which the peak with an RT of 7.57 min was GYM-A, and the other peak was an unknown compound (*m*/*z* 540.4), tentatively named as GYM-K. Moreover, a tiny peak also appeared at 1.50 min (*m*/*z* 524.3), tentatively named GYM-L. Coincidentally, two unknown peaks were also observed in the chromatogram of *m*/*z* 136.1 with a similar RT and corresponding precursor ions to that of *m*/*z* 392.2 (Figure 4B), further proving the existence of both unknown compounds. Subsequently, verification analysis was operated by MRM scan mode (Figure S4). Based on the above results, it was preliminarily speculated that both unknown compounds might be conversion products of GYM-A.

The changes in the relative peak area of GYM-A and both unknown compounds in the samples of pH 7 and 20 °C with storage time are shown in Figure 4C. Both unknown compounds were not detected in the initial solution, but their relative peak area increased with storage duration. After 8 months, the relative peak area of GYM-A decreased to 43.5%, while *m*/*z* 524.3 and *m*/*z* 540.4 increased to 2.5% and 54.0%, respectively. Meanwhile, both unknown compounds were not detected in pH 3 treatment at 20 °C after 8 months (Figure 4D), which was consistent with the results in the stability assessment. Thus, our study suggests that GYM-K and GYM-L are conversion products of GYM-A during storage.

**Figure 5:**
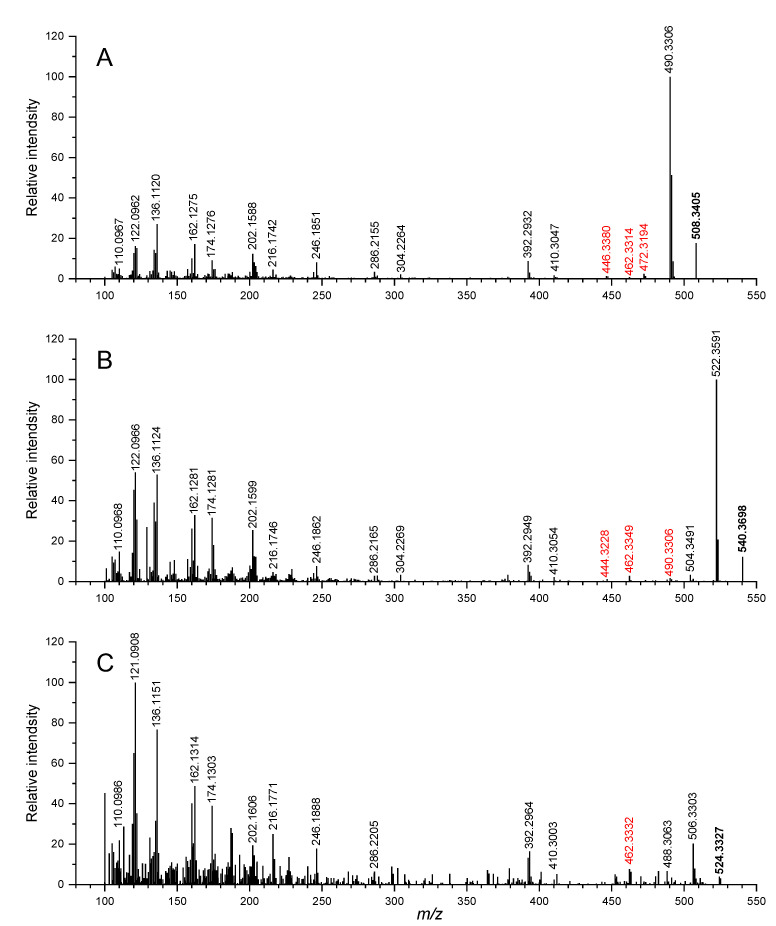
High-resolution product ion spectra of GYM-A parent ion at *m*/*z* 508.3 (**A**) and its conversion products GYM-K (*m*/*z* 540) (**B**) and GYM-L (*m*/*z* 524) (**C**).

In order to further identify the structures of both new compounds, the MS^2^ spectra were acquired by LC-HRMS/MS (Figure 5). The fragmentation spectra generated by the precursor ions at *m*/*z* 524.3327 (C_32_H_46_O_5_N^+^) and *m*/*z* 540.3698 (C_33_H_50_O_5_N^+^) were very close to that of GYM-A, with a lot of overlap, containing the GYM-A characteristic fragments of *m*/*z* 392.2932, 202.1588, 162.1275 and 136.1120 (Figure 5A). Hence, both hypothesis compounds GYM-K and GYM-L were speculated to be structurally related to GYM-A. The main difference in the MS^2^ spectra was the fragment at *m*/*z* 446.3380 in GYM-A versus that at *m*/*z* 462.3349 in both hypothetical compounds.

**Figure 6:**
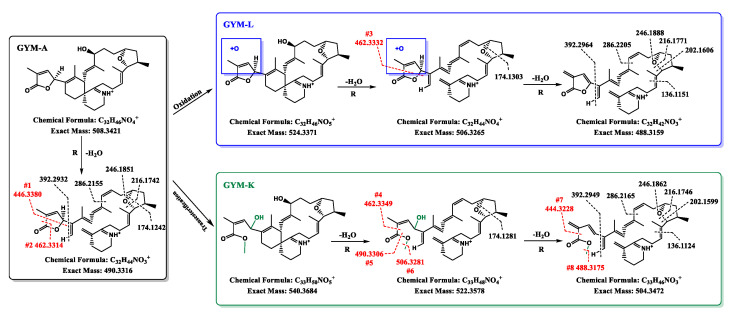
The proposed chemical conversion and fragmentation pathways of GYM-A derivatives. “R” means rearrangement.

Interestingly, the fragments below *m*/*z* 392 in both conversion products were completely consistent with those of the GYM-A (Figure 5), which pointed out that the structure of the product ion at *m*/*z* 392 did not change and the structural change may be present in the butenolide ring moiety [22]. A similar case also occurred in the other analogue of GYMs from shellfish samples, named GYM-F, with an [M + H]^+^ ion at *m*/*z* 526.3529 (C_32_H_48_O_5_N^+^), which has an additional hydroxyl group at C3 and lacks the C2–C3 double bond [23]. In the present study, however, there is no ring-opening reaction or double-bond fracture involved in the conversion to GYM-L because its unsaturation did not change comparatively with GYM-A (Table S7). Additionally, it was noteworthy that the product ion at *m*/*z* 446.3380 (cleavage #1) of GYM-A was not detected in the spectrum of GYM-L (Figure 5C), but a fragment ion at *m*/*z* 462.3332 (cleavage #3) was found. A tiny peak at *m*/*z* 462.3314 was also found in the GYM-A spectrum, but it was formed by cleavage #2 [23]. Coincidentally, GYM-L had the same exact mass as the [M + H]^+^ of GYM-B (Table S7), but this possibility should be ruled out on account of a large difference between their fragment ions (Figure S5). Therefore, the elemental difference between GYM-L and GYM-A (1 O) can be explained by the oxidation of the double bond between C2 and C3 or at C4 (Figure 6) or at C25. Furthermore, GYM-L was not retained on the C18 reversed-phase column (Figure S4C), indicating that it is a highly polar molecule. Regrettably, there was insufficient evidence to confirm which carbon is oxidized from their product ion spectra. Based on the present study, it is hypothesized that GYM-L was formed by the oxidation of the double bond in the butenolide ring of GYM-A.

The case of GYM-K was similar to that of GYM-L, but it may be the main conversion product of GYM-A from the evidence that its relative abundance was higher than GYM-A after 8 months (Figure 4C). As the unsaturation of GYM-K was smaller than that of GYM-A (Table S7), the conversion process from GYM-A to GYM-K may refer to a double-bond fracture or ring opening. The case of a double-bond fracture could be ruled out due to the existence of *m*/*z* 462.3349 (cleavage #4) in GYM-K (Table 2). Most likely, therefore, the butenolide ring was opened at the ester bond position (C4). It has been proven that the ester functionality undergoes ester exchange upon heating in methanol and is accelerated by a base catalyst, and this exchange was called the transesterification (TE) reaction [34]. According to the difference in the elemental formula of GYM-K and GYM-A (1C, 1O, and 4H) (Table S7), the conversion pathway of GYM-K may be the TE reaction of GYM-A with methanol, which brought about the ring opening of the butenolide ring (Figure 6). The fragment ions of GYM-K at *m*/*z* 490.3306 (C_32_H_44_O_3_N^+^) and *m*/*z* 506.3281 (C_32_H_44_O_4_N^+^) due to the cleavage #5 and #6, respectively (Table 2), and the tiny peak after dehydration at cleavage #7 and #8, further strengthen the hypothesis of ring opening of the butenolide ring. A similar TE reaction of ethyl acetate (AcOEt) with methanol has been performed with basic catalysts in the liquid phase, and its activity is directly correlated with the kinetic basicity of the catalysts surface [35]. This conclusion agrees with the result of more transformation of GYM-A at higher pH in this study. Therefore, strong evidence points toward the conversion pathway of GYM-A to GYM-K through the transesterification reaction. Furthermore, okadaic acid (OA) could react with aqueous methanol by enzymatically catalyzed hydrolysis and methanolysis reactions resulting in methylation [36]. Therefore, the conversion pathway of GYM-K may be explained by the transesterification reaction of GYM-A ester functionality with methanol, which caused the methylation and oxidation of GYM-A. The hypothesized product of this transesterification with methanol also explains the similar polarity compared to GYM-A, with a similar retention time of GYM-K compared to GYM-A. However, further studies using labelled methanol for storage would be required to verify whether the conversion follows a pathway of GYM-A via GYM-L to GYM-K or whether transformations are direct from GYM-A to both GYM-L and GYM-K.

To summarize, GYM-A was stable at low pH and temperature in methanol but decreased dramatically stored at 20 °C in high pH solution due to methylation and oxidation. Two hypothesis analogues of GYM-A were identified, namely GYM-K and GYM-L, and their conversion pathways were preliminarily proposed. Unfortunately, the exact structures of both hypothetical analogues were unable to be accurately elucidated by nuclear magnetic resonance (NMR) due to the insufficient quantity. More samples of these hypothesis analogues will be obtained for accurate identification in future studies.

## 3. Conclusions

In the present study, the stability of GYM-A in methanolic media was investigated under varied pH and temperature conditions for an 8-month period. The results show that GYM-A was stable at −20 °C regardless of pH during 8-month storage, while it decreased sharply at pH 5 and 7 under 20 °C. Meanwhile, no significant changes occurred in GYM-A concentration at all temperature levels at low pH (pH 3). Overall, GYM-A was stable at low-temperature (≤−20 °C) and acidic conditions (pH ≤ 3). Additionally, two conversion products of GYM-A, tentatively named GYM-K and GYM-L, were found for the first time. Gymnodimine-L may be formed by the oxidation of GYM-A at the butenolide ring, and GYM-K may be formed by the transesterification reaction of GYM-A ester functionality with methanol. A useful strategy for long-term storage of GYM-A CRM was suggested, and two hypothesis conversion products of GYM-A were found and elucidated in this study. 

## 4. Materials and Methods

### 4.1. Chemicals

Liquid chromatography (LC)-grade methanol and acetonitrile were obtained from Merck KGaA (Darmstadt, HE, Germany). Deionized water (18.2 MΩ cm) was purified by a Milli-Q purification system (Millipore Ltd., Billerica, MA, USA). The CRM of GYM-A was purchased from National Research Council Canada (Halifax, NS, Canada). Sephadex LH−20 was provided by Cytiva (formerly GE Healthcare Life Sciences, Uppsala, Sweden). Acetic acid and ammonium hydroxide (NH_4_OH) were purchased from Fisher Scientific Ltd. (Fair Lawn, NJ, USA).

### 4.2. Biological Source and Preparation of GYM-A

#### 4.2.1. Culture of *Karenia Selliformis*

The GYM-producing dinoflagellate *Karenia selliformis* (strain GM94GAB) [18], isolated from the Gulf of Gabes, Tunisia, was used as the GYM-A source. The strain was cultured in a 15 L photobioreactor (Guangyu Biological Technology Ltd., Shanghai, China) with sterilized seawater filtered by 0.45 μm mixed-fiber membrane (Xingya Ltd., Shanghai, China). The culture was enriched with f/2 medium without silicate [37]. Cultures of *K. selliformis* were grown at 20 ± 2 °C with a 14 h light–10 h dark photoperiod under an approximate photon flux density of 4000 lx cool white light. Microalgal cells were harvested for extraction of GYM-A when they grew in the late exponential growth phase.

#### 4.2.2. Extraction and Purification of GYM-A

The extraction of GYM-A from *K. selliformis* was carried out according to our previous study [30]. In brief, dichloromethane was added to the *K. selliformis* cultures directly as the extractant (55 mL L^−1^), then the organic phase was collected after standing and centrifuged at 4750× *g* for 10 min. The crude extract (containing 65 μg GYM-A) was loaded on a Sephadex LH-20 (16 mm × 700 mm, Cytiva, USA) size exclusion chromatography and eluted by methanol. The fractions containing GYM-A monitored by LC-MS analysis were combined and evaporated with low pressure. The concentrated extract (containing 62 μg GYM-A) was further fractionated by semi-preparative HPLC (Hitachi Primaide, Tokyo, Japan), with a diode array detector (DAD) on 210 nm, on an X-Bridge™ C18 OBD column (250 mm × 10 mm i.d., 5 µm, Waters, Milford, MA, USA) using isocratic elution with 36% B (A: water containing 0.1% acetic acid; B: methanol) at 4 mL min^−1^. The fractions containing GYM-A were combined and then purified using a solid-phase extraction (SPE) cartridge (Oasis HLB, 1 g, Waters, Milford, MA, USA), as described previously [20]. Briefly, the GYM-A fractions were diluted with water to obtain a methanol percent of 20% and loaded on to the SPE cartridges activated and equilibrated in advance, washing with 15 mL 20% methanol to remove the buffer, and eluting with methanol (10 mL). Combination of the purified toxin eluents from several batches of purification process was concentrated and evaluated by a nuclear magnetic resonance (NMR) spectrometer (Pro pulse 500 MHz, Agilent technologies, Wilmington, DE, USA), and then the samples were diluted 1500 times with methanol and separately packed into 247 glass bottles, each bottle containing 2.4 ± 0.12 μg GYM-A. 

### 4.3. Homogeneity Assessment

According to PD ISO GUIDE 35: 2017 [38], ten bottles of the purified GYM-A solutions were randomly selected from the 247 bottles and the homogeneity was evaluated by LC-MS/MS analysis in triplicate. All samples were tested under repetitive conditions in random order, and a QC standard was inserted in the sequence to correct any trends in toxin concentration to be decoupled from instrumental drift. Heterogeneity of toxin concentration was then assessed using one-way analysis of variance (ANOVA).

### 4.4. Stability Assessment 

The long-term stability of GYM-A was assessed by an isochronous evaluation method under different temperature and pH conditions during storage for 8 months. The temperatures of 20 °C (ambient), 4 °C (cold) and −20 °C (freeze) and three levels of pH (3, 5 and 7) were investigated. The pH was adjusted by acetic acid aqueous solution according to the concentration of H^+^ in aqueous medium, as the pH is not defined in organic solvents. A total of 162 samples were kept in the dark and sealed and stored at different experimental conditions. Three subsamples were taken randomly at 0, 16, 60, 120, 180 and 240 d, respectively, and the concentration of GYM-A was quantified by LC-MS/MS.

### 4.5. The LC-MS/MS Analysis Method 

The LC-MS/MS analysis was performed using an Ultimate 3000 UPLC (Thermo Fisher Scientific, Bremen, Germany) coupled to an AB-Sciex Qtrap 4500 quadrupole mass spectrometer (AB Sciex Pte. Ltd., Singapore City, Singapore) equipped with an electrospray ionization (ESI) interface. Chromatographic separation was carried out on an X-Bridge™ C18 reversed-phase column (150 mm × 3 mm, 5 μm, Waters, Milford, MA, USA) using a binary mobile phase of solvent A (water) and B (90% acetonitrile/water), both containing 6.7 mmol L^−1^ ammonium hydroxide. Gradient elution consisted of 50% to 100% B over 8 min with 2 min hold at 100% B, and returned to 50% B over 1 min before re-equilibration for 1 min at 0.3 mL min^−1^ with the column at 35 °C. Multiple reaction monitoring (MRM) mode with positive ionization was used for all quantitative measurements, and the acquisition parameters are shown in Table 3. A GYM-A CRM from NRC was used as the external standard for calibration of LC-MS measurements, and the linear range of GYM-A was 2.1 to 40 ng mL^−1^ (Figure S6). The identification of unknown products was implemented by precursor ion scan mode using the product ions of *m*/*z* 392.2 and 136.1, where *m*/*z* 392.2 is commonly used as the GYM-A qualitative ion and *m*/*z* 136.1 is the characteristic fragment of all analogues of GYMs.

### 4.6. The LC-HRMS/MS Analysis

The LC-HRMS/MS analysis was carried out on a high-resolution time-of-flight mass spectrometer (Q-TOF 6546 iFunnel, Agilent technologies, Wilmington, DE, USA) equipped with a Dual AJS electrospray ionization (ESI) coupled with an Agilent 1290 Infinity II UHPLC system (Agilent technologies, Wilmington, DE, USA). The chromatographic separation parameters were the same as LC-MS/MS analysis. The acquisition range for MS and MS^2^ were set from *m*/*z* 100 to 1700. Mass spectral acquisition was performed in positive (ESI ^+^) ion mode. The ESI source parameters were set as follows: sheath gas temperature, 350 °C; sheath gas flow rate, 11 L min^−1^; nebulizer pressure (N_2_), 40 psi; capillary voltage, 4.0 kV; fragmentor voltage, 155 V; and skimmer voltage, 65 V.

### 4.7. Statistical Analysis

The GYM-A homogeneity data were statistically analyzed by one-way analysis of variance (ANOVA) according to ISO GUIDE 35: 2017. Stability data were expressed as mean ± standard deviation (SD) and fitted by linear regression, then the significance of linear regression slope was tested by *t*-test. Statistical analysis was performed on SPSS Statistics 25 (IBM, Armonk, NY, USA). The LC-HRMS/MS data were processed by Qualitative Navigator software (version B.08, Agilent Technologies, CA, USA). All figures were drawn by the Origin 2019b package (Origin Lab, Hampton, MA, USA).

## Figures and Tables

**Table 1:** The *t*-test results of linear regression slope of GYM-A stability under different pH and temperature levels during 8-month storage.

Treatments		Slope	Standard Error	*t*	*p*	Stability
−20 ℃	pH 3	−0.0047	0.0028	−1.685	0.167	Stable
	pH 5	−0.0062	0.0026	−2.346	0.079	Stable
	pH 7	−0.0077	0.0035	−2.226	0.090	Stable
4 ℃	pH 3	−0.0084	0.0041	−2.023	0.113	Stable
	pH 5	−0.0123	0.0043	−2.845	0.047	Unstable
	pH 7	−0.0207	0.0071	−2.925	0.043	Unstable
20 ℃	pH 3	−0.0149	0.0057	−2.600	0.060	Stable
	pH 5	−0.0839	0.0112	−7.501	0.002	Unstable
	pH 7	−0.1170	0.0124	−9.462	< 0.001	Unstable

**Table 2:** Measured accurate masses and theoretical exact masses for [M + H]^+^ and product ions of *m*/*z* 540 (retention time of 8.37 min).

Fragment Ions	Molecular Formula	Theoretical Exact Mass (*m*/*z*)	Measured Exact Mass (*m*/*z*)	Δ ppm	Relative Abundance (%)
[M + H]^+^	C_33_H_50_O_5_N^+^	540.3684	540.3698	2.59	12.3
[M + H − H_2_O]^+^	C_33_H_48_O_4_N^+^	522.3578	522.3591	2.49	100.0
[M + H − 2H_2_O]^+^	C_32_H_46_O_3_N^+^	504.3472	504.3491	3.77	3.4
	C_8_H_12_N^+^	122.0964	122.0966	1.64	54.0
	C_9_H_14_N^+^	136.1121	136.1124	2.20	52.9
	C_11_H_14_N^+^	162.1277	162.1281	2.47	32.9
	C_12_H_16_N^+^	174.1277	174.1281	2.30	31.5
	C_14_H_20_N^+^	202.1590	202.1599	4.45	25.4
	C_15_H_22_N^+^	216.1747	216.1746	−0.46	4.7
	C_16_H_24_ON^+^	246.1853	246.1862	3.66	7.7
	C_19_H_30_O_2_N^+^	304.2271	304.2269	−0.66	3.3
	C_27_H_38_ON^+^	392.2948	392.2949	0.25	8.3
	C_27_H_40_O_2_N^+^	410.3054	410.3054	0.00	2.2
	C_31_H_42_ON^+^	444.3261	444.3228	−7.43	0.4
	C_31_H_44_O_2_N^+^	462.3367	462.3349	−3.89	2.8
	C_32_H_42_O_3_N^+^	488.3159	488.3175	3.28	0.9
	C_32_H_44_O_3_N^+^	490.3316	490.3306	−2.04	1.7
	C_32_H_44_O_4_N^+^	506.3265	506.3281	3.16	1.3

**Table 3:** Acquisition parameters of multiple reaction monitoring (MRM) mode scanning for GYM-A.

ESI mode	Precursor ions [M + H]^+^ (*m*/*z*)	Product ions (*m*/*z*)	Fragmentor (V)	Collision Energy (eV)
ESI^+^	508.3	490.3	55	45
		392.2	55	50

## Data Availability

The data in this study are available in this article and Supplementary Materials.

## Supplementary Materials

The following supporting information can be downloaded at: https://www.mdpi.com/article/10.3390/toxins14110744/s1, Figure S1: Observed relative concentrations of GYM-A CRMs in the homogeneity assessment; Figure S2: Full-scan chromatogram of the purified GYM-A CRMs (*m*/*z* 50-1500); Figure S3: ^1^H NMR spectrum (0.5–7.5 ppm) of GYM-A in CD_3_OD; Figure S4: Extracted ion chromatograms (XIC) of GYM-A (A), GYM-K (*m*/*z* 540.4) (B), and GYM-L (*m*/*z* 524.3) (C); Figure S5: High-resolution product ion spectra of GYM-B at *m*/*z* 524.3365 (A) and GYM-L at *m*/*z* 524.3327 (B); Figure S6: Calibration curve of GYM-A quantification by LC-MS/MS; Table S1: Measurement data of homogeneity assessment of GYM-A (μg mL^−1^); Table S2: The ANOVA analysis of homogeneity assessment; Table S3: The qNMR analysis results of GYM-A purity assessment; Table S4: The solvent volume of GYM-A samples under different treatments after 4 months; Table S5: The solvent volume of GYM-A samples under different treatments after 6 months; Table S6: The solvent volume of GYM-A samples under different treatments after 8 months; Table S7: List of the compounds corresponding to gymnodimines in this study. Mass differences (Δ ppm) were compared between measured and exact theoretical masses.
