# Supplementary material for: Stability and Chemical Conversion of the Purified Reference Material of Gymnodimine-A under Different Temperature and pH Conditions
Source: Toxins (Basel). 2022 Oct 29;14(11):744. doi: 10.3390/toxins14110744 (PMC9695126; doi:10.3390/toxins14110744)
Supplement: Supplementary file 1 [file toxins-14-00744-s001.zip › toxins-2006453-supplementary.pdf]

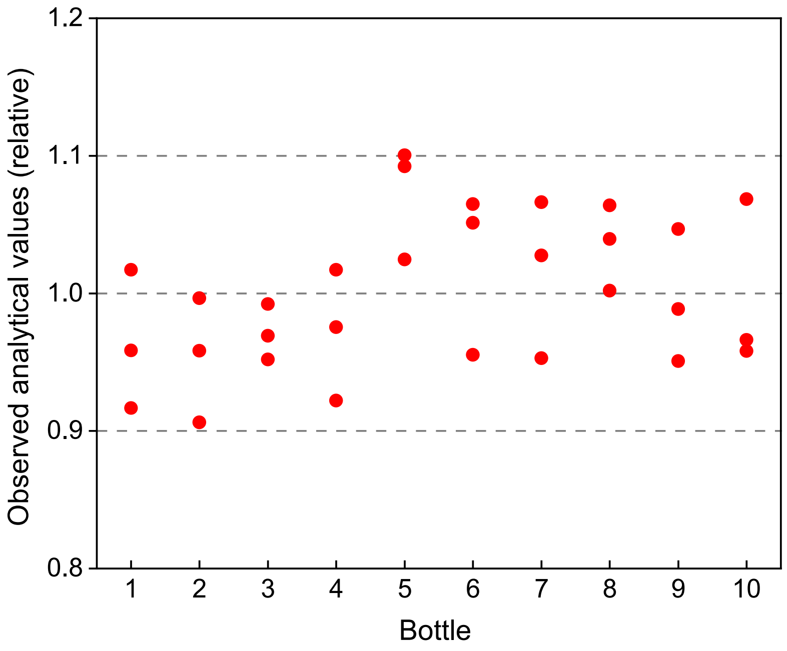

Figure S1. Observed relative concentrations of GYM-A CRMs in the homogeneity assessment.

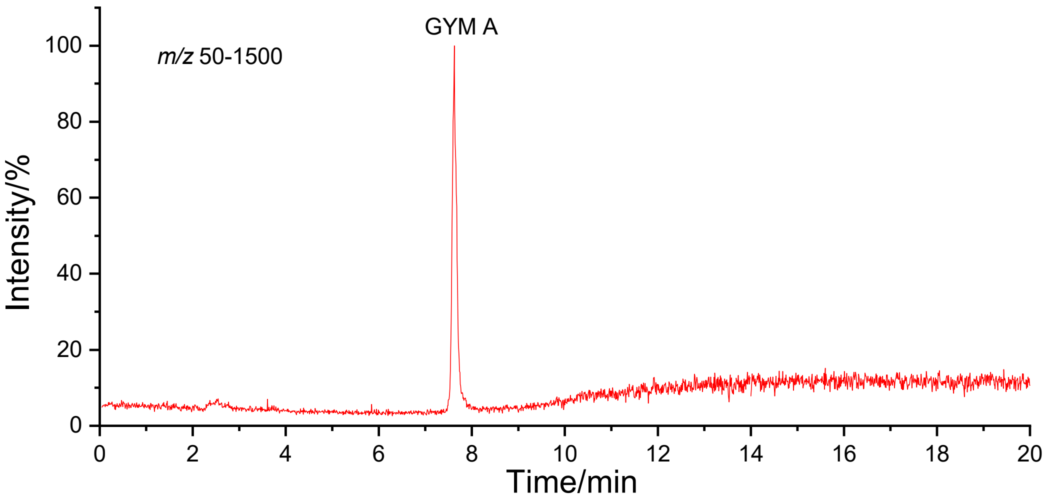

Figure S2. Full-scan chromatogram of the purified GYM-A CRMs (*m*/*z* 50-1500).

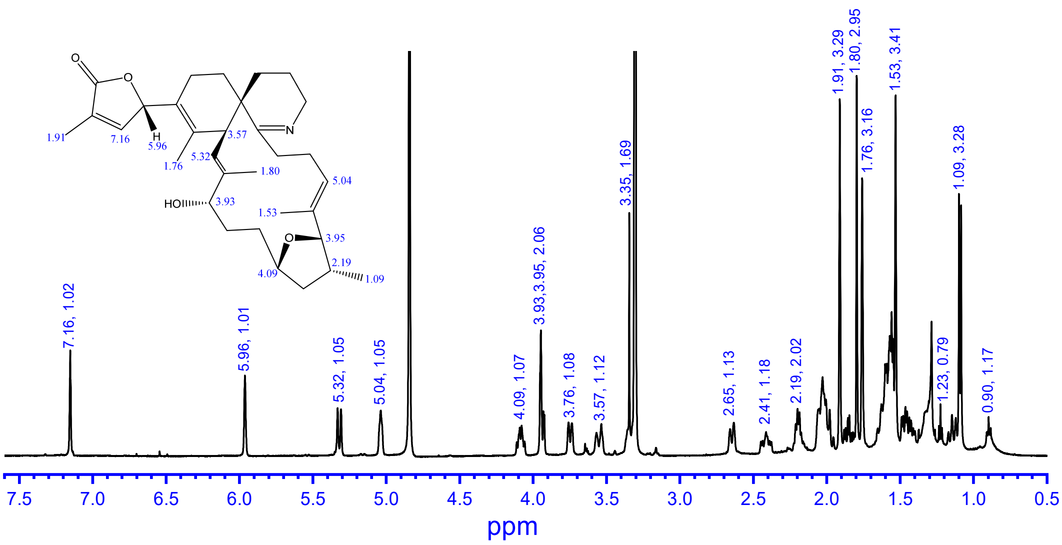

Figure S3. ^{1}H NMR spectrum (0.5–7.5 ppm) of GYM-A in CD_3OD.

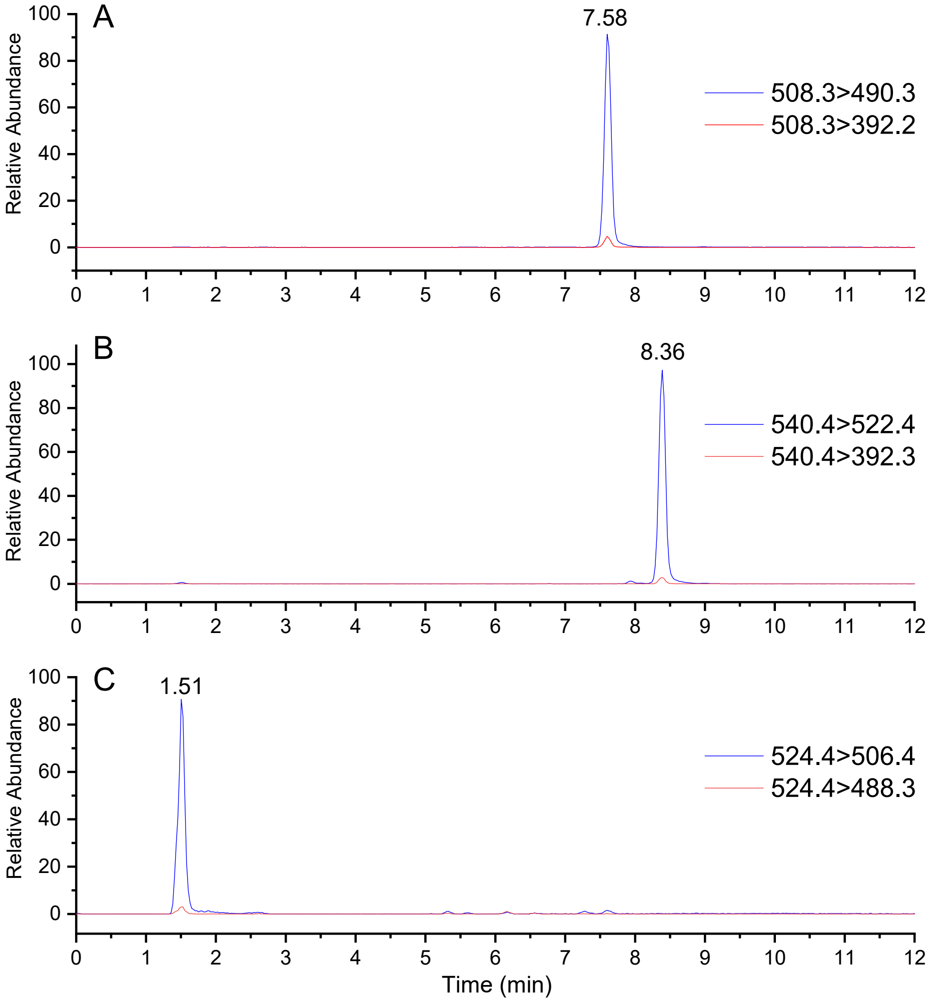

Figure S4. Extracted ion chromatograms (XIC) of GYM-A (**A**), GYM-K (*m/z* 540.4) (**B**), and GYM-L (*m/z* 524.3) (**C**).

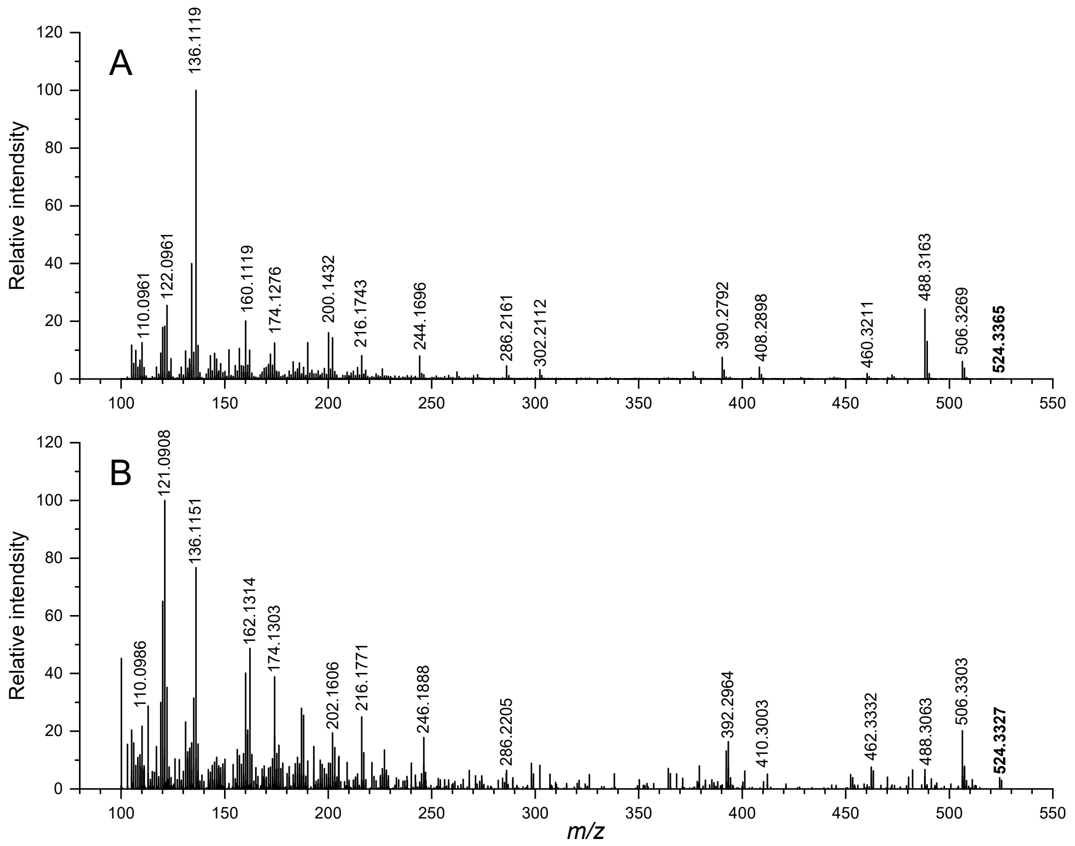

Figure S5. High-resolution product ion spectra of GYM-B at *m*/*z* 524.3365 (**A**) and GYM-L at *m*/*z* 524.3327 (**B**).

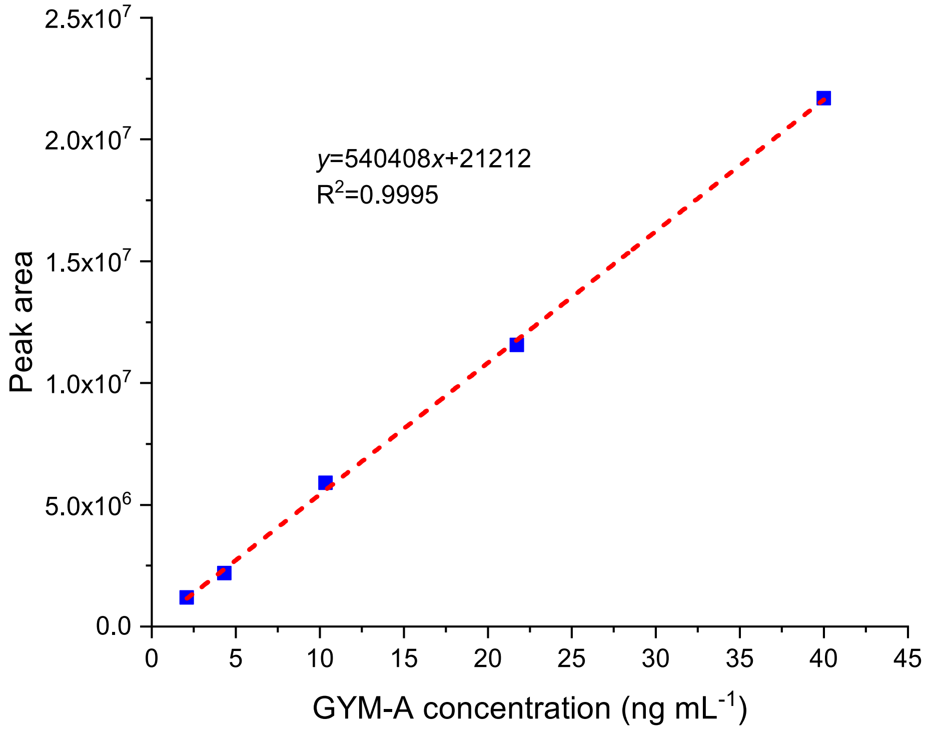

Figure S6. Calibration curve of GYM-A quantification by LC-MS/MS

Table S1. Measurement data of homogeneity assessment of GYM-A (μg mL^{-1})

**Bottle No.**	**Run 1**	**Run 2**	**Run 3**	**Mean**	**Standard error**
1	0.569	0.544	0.604	0.572	0.017
2	0.538	0.592	0.569	0.566	0.016
3	0.565	0.589	0.575	0.577	0.007
4	0.548	0.604	0.579	0.577	0.016
5	0.608	0.649	0.653	0.637	0.014
6	0.567	0.624	0.632	0.608	0.020
7	0.566	0.633	0.610	0.603	0.020
8	0.595	0.617	0.632	0.615	0.011
9	0.565	0.622	0.587	0.591	0.017
10	0.574	0.569	0.634	0.592	0.021

Table S2. The ANOVA analysis of homogeneity assessment.

**Source of variation**	**Sum of squares**	**Degree of freedom**	**Mean square**
Between bottles	0.013	9	0.0014 (s_1^2)
Within bottles	0.016	20	0.0008 (s_2^2)
Total	0.029	29	

Table S3. The qNMR analysis results of GYM-A purity assessment.

Toxin	Weighing result (mg)	qNMR result (mg)	Purity (%)
GYM-A	1.954	1.868	95.6

Table S4. The solvent volume of GYM-A samples under different treatments after 4 months.

120 d	Temperature (°C)	Solvent volume (μL)			Mean	Standard error
		1	2	3		
pH 3	20	80.9	69.3	68.4	72.9	4.0
	4	90.7	91.6	85.4	89.2	2.0
	-20	87.1	82.7	83.6	84.5	1.4
pH 5	20	69.3	75.5	64.8	69.9	3.1
	4	92.5	94.3	93.4	93.4	0.5
	-20	92.5	93.4	90.7	92.2	0.8
pH 7	20	55.0	84.5	70.2	69.9	8.5
	4	91.6	88.9	85.4	88.6	1.8
	-20	87.1	89.8	84.5	87.1	1.5

Table S5. The solvent volume of GYM-A samples under different treatments after 6 months.

180 d	Temperature (°C)	Solvent volume (μL)			Mean	Standard error
		1	2	3		
pH 3	20	70.2	73.8	67.5	70.5	1.8
	4	81.8	85.4	79.1	82.1	1.8
	-20	84.5	87.1	80.9	84.2	1.8
pH 5	20	88.0	67.5	77.3	77.6	5.9
	4	88.0	91.6	93.4	91.0	1.6
	-20	99.6	83.6	86.3	89.8	5.0
pH 7	20	46.1	79.1	63.9	63.0	9.5
	4	69.3	92.5	89.8	83.9	7.3
	-20	89.8	80.9	95.2	88.6	4.2

Table S6. The solvent volume of GYM-A samples under different treatments after 8 months.

240 d	Temperature (°C)	solvent volume (μL)			Mean	Standard error
		1	2	3		
pH 3	20	75.0	67.0	43.8	61.9	9.4
	4	87.5	75.0	91.1	84.5	4.9
	-20	89.8	91.6	72.9	84.8	6.0
pH 5	20	85.7	84.8	42.0	70.8	14.4
	4	91.1	81.3	75.9	82.7	4.4
	-20	89.8	83.6	91.6	88.3	2.4
pH 7	20	70.5	72.3	38.4	60.4	11.0
	4	91.1	88.4	91.1	90.2	0.9
	-20	78.2	81.8	84.5	81.5	1.8

Table S7. List of the compounds corresponding to gymnodimines in this study. Mass differences (Δ ppm) were compared between measured and exact theoretical masses.

**Compound**	**RT (min)**	**Molecular formula**	**[M+H]⁺ theoretical *m/z***	**Δppm**	**Unsaturation**	**Putative identity**
**Compound 1 524.3327**	1.51	$C_{32}H_{45}NO_5$	524.3371	-8.392	11	GYM-L
**Compound 2 508.3405**	7.58	$C_{32}H_{45}NO_4$	508.3421	-3.147	11	GYM-A
**Compound 3 540.3698**	8.36	$C_{33}H_{49}NO_5$	540.3684	2.591	10	GYM-K
